# Assessment of the impact of innovative fertilization methods compared to traditional fertilization in the cultivation of highbush blueberry

**DOI:** 10.1371/journal.pone.0271383

**Published:** 2022-07-20

**Authors:** Agnieszka Lenart, Dariusz Wrona, Kamila Klimek, Magdalena Kapłan, Tomasz Krupa

**Affiliations:** 1 Department of Pomology and Horticultural Economics, Warsaw University of Life Sciences—SGGW, Belsk Duży, Poland; 2 Department of Applied Mathematics and Computer Science, University of Life Sciences in Lublin, Lublin, Poland; 3 Department of Pomology, Nursery and Enology, University of Life Sciences in Lublin, Lublin, Poland; Bahauddin Zakariya University, PAKISTAN

## Abstract

The aim of the research was to evaluate fertilization technologies for the indicators of the quality and quantity of highbush blueberry yield. In the experiment, a similar level of mineral fertilization was used in all treatments. The experiment was to show the differences between fertilization with biostimulation and without biostimulation. The research was carried out in two seasons (2019–2020) on ´Bluecropˋ shrubs growing in the Blueberry Experimental Field in central Poland (51° 55’42.7 "N 20° 59’28.7" E). Shrubs grow at a distance of 1 x 3 m. Plants are rejuvenated every year in spring and irrigated by drip. The experiment was carried out in a random block design (4 fertilizer treatments x 5 replications x 6 bushes). The experiment assessed the effect of fertilization on yield, berry mass, fruit setting, leaf surface and physicochemical parameters of fruit. Based on the conducted research, it was proved that the applied fertilization technologies had a significant impact on the size and quality of the yield of “Bluecrop” highbush blueberry. Particularly noteworthy is the fertilization technology with biostimulation (treatment T4), which has a positive effect on the yield, fruit mass, percentage of setting and firmness of the berries. Analysis of the issue in the light of the results of the conducted research shows that the use of biostimulated products has an important impact on the intensification of production while maintaining good quality of fruits. Through research, the positive effect of fertilization programs with biostimulation (treatment T4) on the most important production parameters of blueberry fruit from the producer’s point of view has been proven.

## Introduction

Constantly growing consumer demand for blueberry fruit makes the cultivation of this species expand, year by year, in Poland and around the world. The high demand for highbush blueberry fruit is driven by its pro-health and taste values, as well as a wide-ranging campaign promoting blueberry as superfoods. According to the International Blueberry Organization, the production of blueberries in 2020 exceeded 1 million tons worldwide [1]. In recent years, the agricultural sector has faced the challenges of increasing production in order to feed a growing world population and use resources efficiently, while reducing the impact of agricultural production on ecosystems and human health. Fertilizers and pesticides play a key role in agriculture, providing growers with a tool to increase and ensure high-quality yield, both under optimal and stressful conditions [2]. An innovative and environmentally friendly solution is the use of natural plant biostimulants, which support flowering, plant growth, fruit setting, yielding and nutrient use efficiency, as well as increasing tolerance to a wide range of abiotic stressors [3]. Substances containing seaweed increase the tolerance to abiotic stress, and improve plant performance and durability of the fruit, so unique chemical composition of marine algae is associated with the environment in which they live (salt water, low temperatures, lack of light, the periodic ebb and drying) [4, 5].

Of particular interest are fertilizers with biostimulation that are using extracts of marine algae. The complex chemical composition of seaweed extracts containing valuable nutrients and biostimulants, i.e. macro and microelements, amino acids, vitamins, cytokinins, auxins, abscisic acid (ABA) [6–9], determines their way of interaction in plants. The great advantage of marine algae is their growth power, which is ten times greater than that of maize. About 250 tons of *Laminaria Digitata* algae are obtained from one hectare of the sea surface, while about 22 tons of dry matter of maize from one hectare of land [10]. Although marine algae have been used in agricultural production for decades and the first records of their use as a fertilizer are dated as late as 16th century, [11–13] there is still no legal definition of biostimulation or biostimulant. Defining the biological basis of biostimulants as a class of compounds complicates the variety of biostimulants available on the market. These include bacteria, fungi, sea algae extracts, telomic plant extracts, raw materials of animal origin. The same is true for the variety of industrial processes implemented for the preparation of biostimulant products [14]. Du Jardin (2015) [15] proposed the following definition: "A plant biostimulant is any substance or microorganism applied to plants with the aim to enhance nutrition efficiency, abiotic stress tolerance and / or crop quality traits, regardless of its nutrients content. By extension, plant biostimulants also designate commercial products containing mixtures of such substances and / or microorganisms". Based on this definition, we assume that biostimulants have a beneficial effect on crop productivity by interacting with plant physiological processes, increasing plant resistance to stress [16]. Biostimulation in agriculture has been the subject of many recent studies showing that plants’ stress response is regulated by signaling molecules produced by the plant or related microorganisms [17–19]. Biostimulants can either directly interact with signaling molecules generated by plants or stimulate plant-related and beneficial microorganisms [16]. From the perspective of growing consumer demands, with the changing availability of plant protection products and changing climatic conditions, stimulating the natural resistance and yield of plants using natural bioactive substances [20] is the most effective method of building both the quantity and quality of the crop. Compounds contained in marine algae, not only have a positive effect on the soil structure and its water capacity [21], but also stimulate the development of beneficial soil microorganisms [13]. Plants treated with sea algae extracts take up and assimilate nutrients faster than untreated plants [22], are characterized by stronger growth [13, 22] and have a well-developed root system with numerous fine side roots [23, 24]. Calvo et al., 2014; Rose et al., 2014 [25, 26] provide evidence that biostimulants can increase macronutrient uptake and have been attributed to an effect on the absorption activity or stimulation of nitrogen metabolism. Also, Saa et al., (2015) [27] report that in the experiment with almonds grown in conditions of high nutrient supply, sea algae extracts or products of microbial fermentation of cereal grains clearly had a positive effect on shoot growth and leaf surface. Algae extracts have a positive effect on the effectiveness of plant protection against diseases and pests [28, 29], increase tolerance to drought [30] and high temperature [31–33].

In addition to proper mineral fertilization, biostimulants can increase the effectiveness of conventional fertilizers [34], absorb and accumulate greater amounts of macronutrients at the leaf level [22]. In the studies of Mancuso et al [22], the IPA extract (Adenine Isopentyl) had an effective impact on the accumulation of nitrogen, phosphorus and potassium in grapevine plants. The role of biostimulants in the accumulation of nutrients at the tissue level is still under investigation. According to Salat, [35] biostimulators may contain chelating agents (e.g. mannitol in seaweed), which can enhance the availability of nutrients and a better absorption of the chelate from the surface of the leaves. In this manuscript, the authors assessed the impact of fertilization technology on the quality and yield of blueberries. The aim of the study was to show the differences between fertilization with and without biostimulation. The purpose of the research is new, and the products used for the research are innovative. The conducted research provides knowledge on the use of fertilization with biostimulation in blueberry cultivation.

## Research methodology

This work was carried out under the program of the Ministry of Science and Higher Education "Doktorat Wdrożeniowy" no. um. 0060 / DW / 2018/02. The aim of the program is to create conditions for the development of cooperation between the scientific community and the socio-economic community conducted as part of doctoral studies, introducing the possibility of educating a participant of doctoral studies in cooperation with the entrepreneur (or other entity) employing him/her. The research was carried out in 2019–2020 at the Experimental Blueberry Field, Warsaw University of Life Sciences in Błonie near Prażmów, central Poland (51° 55’42.7 "N 20° 59’28.7" E). More than 30-year-old shrubs of the ´Bluecropˋ cultivar grow at a spacing of 1 x 3 m. In accordance with IPO practices, plant protection treatment like rejuvenating pruning is carried out in the quarters, and in addition to that, the quarters are irrigated by drip. The pH range of the substrate during the experiment was between 4,5 and 4,8. In the experiment, the soil was tested in a certified laboratory of the Regional Chemical and Agricultural Station in Łódź (Table 1) and, on account of the results, nutrients were supplemented to optimal values (Table 2). Permission was obtained from Warsaw University of Life Science to collect plant materials and all study/experimental protocols involving plant materials were conducted in accordance with institutional, national, and international guidelines and legislation.

**Table 1:** Test report no. GO / 502/18.

Code	Customer’s sample labelling	Salinity	pH	Content in mg / l						
letter-digit sample		g NaCl / l	in H_2_O	N-NO_3_	N-NH_4_	P	K	Ca	Mg	Cl
**GO / 502/4/18**	**4**	0.08	4.9	<10.0 *	<10.0 *	<20.0 *	<20.0 *	245	20	<10.0 *
Research Procedure / Standard		PB 02 ed. 3 from March 1, 2018	PB 01 ed.2 from March 1 2018	PB 06 ed.1 from May 28, 2004	PB 69 ed.1 from April 3 2017	PB 03 ed.2 from March 19, 2007	PB 04 ed.1 from May 21, 2004	PB 04 ed.1 from May 21, 2004	PB 05 ed.1 from May 28, 2004	PB 07 ed.1 from May 28, 2004

* /—result below the lower range of the method.

**Table 2:** Sum of nutrients used in the experiment in all assessed treatment (K,T,M,W).

N [kg / ha^-1^]	P_2_O_5_ [kg / ha^-1^]	K_2_O [kg / ha^-1^]	SO_3_ [kg / ha^-1^]	CaCO_3_ [kg / ha^-1^]
100	30	92.5	142	64

The research material consisted of shrubs and blueberries of ´Bluecropˋ highbush cultivar. The experiment was carried out in a random block system. Four fertilizer treatments were tested, with five repetitions in each treatment. Each replicate contained six plants. In 2019, the harvest was carried out from 01.07.2019 to 10.08.2019 and in 2020 from 05.07.2020 to 05.08.2020. Harvested fruit was a collective sample, averaged from all harvests. The experiment assessed the effect of biostimulation on yield, mass of 100 berries, fruit setting, leaf surface and quality parameters of fruit.

- ’Treatment T1’—included traditional sprinkling and foliar fertilization without bioactive substances (control treatment).
- ’Treatment T2’—included traditional sprinkling and foliar fertilization extended with a preparation containing phytohormone precursors and biostimulants.
- ’Treatment T3’—included traditional sprinkling and foliar fertilization extended with an implementation preparation (currently ongoing registration studies) containing bioactive substances.
- ’Treatment T4’—soil and foliar fertilization with preparations containing biostimulation was applied in treatment.

In the performed experiment, in each treatment, the amount of nutrients supplied to the plants (N, K, P, Mg etc.) was equal or very close. The assessed factor was the method of fertilization and active compounds (biostimulants) occurring in various forms and concentrations.

In treatment T1 traditional sprinkled and foliar mineral fertilizers available on the market were used (Table 2), containing no additional bioactive or anti-stress substances. Combination T1 is a control where only mineral fertilization was used, without biostimulation.

Treatment T2 used a biostimulating product (N 3%, P 7%, K 7%, Mn 0,05%, Zn 0,1%) with complex NMX^®^ (Patent No. EP 01500090.4) It is a growth stimulating composition for plants characterized by one or several of the following components: a precursor compound of cyclic AMP (cyclic adenosine monophosphate) for its transformation in the latter compound in the interior of the cells, a compound with the capacity to inhibit the activity of the enzymes of the phosphodiesterases family, a compound with the capacity to stimulate the activity of the enzymes of the Adenyl- Cyclase family, an agonist compound of the β-adrenergic receptors, a chosen compound between arachidonic acid or a prostaglandin. According to the producer, this product is a natural enhancement of all plants’ processes involved in fruit setting, complete and sustainable action to support the plant from fruit setting to fruit ripening, reinforcement of the development of the fruits coming from the parthenocarpic.

In treatment T4, the full biostimulation program recommended by the manufacturer of fertilizers based on marine algae extracts was used. The complex NMX® was used as in treatment M, and biostimulating complex Fertiactyl^®^ (patent number 945000107) combining 3 active substances (humic and fulvic acids, glycine-betaine, zeatine) and mineral elements (N 13%, P 5%, K 8%) that work in synergy. According to the producer, it improves abiotic stress resistance to external pressures (heat stress and drought stress). When the stress occurs, glycine-betaine complex stabilizes the balance of water / mineral salts in the leaves, enabling them to keep working. Biostimulation complex improves photosynthesis by protecting the chloroplasts from aging, naturally stimulates the development of new organs rooting and tillering. Additionally, the Seactiv^®^ complex (Patent No. EP98400150.3)—contains seaweed extracts and mineral nutrients, whereas the main components are a hormonal precursor like isopentyl of adenine, natural osmolytes (glycine—betaine) and amino-acids. According to the producer, the complex of phytohormones, increases plant’s tolerance to stresses whilst boosting its physiological processes. It also enhances root development, nutrient absorption and nutrient movement within the plant, as well as increases the homogeneity (evenness) of crops through delayed senescence "Stay Green Effect". What is more, leaf photosynthesis is optimized, while also ensuring homogenous fertilization, flowering and quality of fruit / grain yield. Also, soil fertilization was based on a preparation with biostimulation. Nprocess^®^ complex (NPK 8-8-17, MgO 3%, SO_3_ 29%, CaCO_3_ 14%, B 0,15%, Zn 0,1%)—allows a constant flow of nitrate with increased absorption (“pump effect”), improves nitrogen transformation within the plant, therefore generating more protein and Dry Matter (DM). N-PRO is an Indolic hormone which enhances the action of nitrate reductase and stimulates crop demand for nitrogen, which is essential for fast and effective conversion of nitrates into useable plant proteins. The Top–Phos^®^ complex (NPK 5-10-15, CaO 10%, MgO 3%, SO_3_ 37%, B 0,15%, Zn 0,1%) used, is a new molecule of phosphorus that contains an organic matrix being immediately available for plant uptake throughout the growing season. Phosphate availability is combined with a root system stimulator and a biological activity booster.

In treatment T3, a new type of biostimulation was used, based on extracts from the Kaori tree and sea algae, with bioactive properties, aimed at improving the physiological processes in crops (preparation under registration studies).

Both the yield of the experiment and the mass of 100 fruits were assessed based on the unit mass of the fruit harvested within the replication. Measurements were made using a precise digital scale Elegance (Höffman, Braunschweig, Germany). Assessment of setting was performed for each treatment on 15 representative perennial shoots (100 flowers were counted in 12 replications for each treatment). Flowers were counted at the beginning of flowering, then fruit buds were counted 21 days after flowering. Based on the number of flowers in relation to the number of fruits, the degree of fruit set was calculated. Leaf area was assessed on the grounds of 1.200 representative leaves within each treatment using the 3100 Area Meter (PG Debrunner Ing, Bad Homburg, Germany).

Fruit acidity was determined by titration according to PN-EN 12147: 2000 [36] standard. To determine the acidity, 20 fresh fruits from each replication were used. The juice for the test was obtained by crushing the fruit with a DI 25 Basic mill (Kika–Werke GMBH and CO, Staufen, Germany) and then the obtained homogenate was centrifuged for 10 minutes at 2° C. The juice was mixed with distilled water in a ratio of 1:10 (v:v) and titrated with 0,1 M NaOH to pH 8.1 using TitroLine 5000 (Si Analytics, Mainz, Germany). The amount of NaOH consumed was then converted to the percentage of citric acid.

Fruit firmness was measured according to the method described by Szpadzik et al [37] on 20 freshly picked fruits from each repetition. The INSTRON 5542 firmness gauge was used for the analyzes (Instron Corporation, Norwood, Massachusetts USA). A 4.5 mm diameter pin was used to measure fruit firmness by inserting the pin 5 mm into the fruit pulp. Measurements were made once on each fruit in the vertical part. The results are given in Newtons.

The content of the extract was determined in accordance with the PN-EN 12143: 2000 [38] standard in the fruit juice of each repetition. The analysis of the extract content was performed using a PR-32 ALPHA digital refractometer (Atago, Tokyo, Japan). Clear juice for the test was obtained the same way as in the measurement of titratable acidity.

### Statistical analysis

Test results were analyzed statistically using the one-way analysis of variance method. The inference was based on the significance level <0.05. All statistical analyzes were performed in the SAS Enterprise Guide 5.1 program (Sas Institute Sp. z o.o., Warsaw, Poland).

## Results

The yield of ´Bluecropˋ blueberry fluctuated from 4.2 to 5.9 kg^.^shrub^-1,^ i.e. from 14.1 to 19.5 t^.^ha^-1^ and significantly depended on the fertilization technology used and the year of research (Table 3). Shrubs from treatment W yielded significantly lower than those with technology with biostimulation from treatment T. Regardless of the treatment used, in 2020, the plants yielded significantly better than in 2019. Statistical analysis showed a significant impact of the applied fertilization technologies on the mass of highbush blueberries (Table 3). It was found that the bushes fertilized with the biostimulation technology (T4) had a significantly higher mass of 100 berries than the control bushes (T1). There was no significant influence of the study year on the trait studied. The setting degree of “Bluecrop” highbush blueberry was significantly modified by the fertilization technology (Table 3). The bushes treated with biostimulation preparations (T4) were characterized by a significantly higher level of fruit setting than those sprayed with the implementation preparation (T3). There was no significant influence of the study year on the trait studied. The leaf area of highbush blueberry shrubs ranged from 21.5 to 22.7 cm^2^ and did not differ significantly between the treatments used and the years of research (Table 3). The level of acidity of blueberry fruits was significantly influenced by the fertilization technology, the fruits from the shrubs sprayed with the implementation preparation (T3) were characterized by a significantly lower level of acidity than the control ones and treated with the biostimulation technology (T4) (Table 3). There was no significant influence of the study year on the trait studied. The interaction of the fertilization technology and the research year was significant for the analyzed parameter. Blueberry fruit extract ranged from 11.6 to 12.1% and did not differ significantly between the assessed treatments (Table 3). A substantial influence of the study year on the assessed fruit quality parameter was demonstrated. In the first year of the study, blueberries were characterized by a significantly higher level of extract than in 2020. The fruit firmness significantly depended on the fertilization technology used, the shrubs treated with the biostimulation technology (T4) had significantly firmer berries than the control berries (T1) (Table 3). A major impact of the study year on the assessed fruit quality parameter was demonstrated, in 2019 blueberries were notably less firm than in 2020 (Table 3).

**Table 3:** Influence of fertilization technology on the yield and quality of ´Bluecropˋ highbush blueberry.

	Average yield per bush [kg / bush^-1]^	Average yield per hectare [t / ha^-1]^	Mass of 100 berries [g]	Average fruit setting, [%]	Leaf area [cm^2^]	Acidity [% citric acid]	Soluble solids [Brix °]	Firmness [N]
T1 -Control	5.1 ± 1.5 AB	16.9 ± 5.1 AB	200.1 ± 13.9 B	83.7 ± 11 AB	21.5 ± 5.2 A	0.63 ± 0.05 B	11.8 ± 1.2 A	3.5 ± 0.3 B
T4—Technology with biostimulation	5.9 ± 2.5 A	19.5 ± 8.2 A	224.7 ± 13.9 A	93.8 ± 7 A	22.7 ± 0.8 A	0.59 ± 0.12 B	12.1 ± 1.1 A	3.8 ± 0.3 A
T2- Preparation with hormone precursors	4.8 ± 1.2 AB	15.85 ± 3.8 AB	209.5 ± 9.9 AB	88.7 ± 4 AB	21.7 ± 1, 4 A	0.65 ± 0.07 AB	12.1 ± 1.3 A	3.7 ± 0.3 AB
T3—Implementation preparation	4.2 ± 0.6 B	14.1 ± 2.1 B	210.23 ± 16.3 AB	83.3 ± 11 B	21.9 ± 1.5 A	0.73 ± 0.11 A	11.6 ± 1.5 A	3.7 ± 0.3 AB
*p-value*	***0*.*0574***	***0*, *0574***	***0*.*0047***	***0*.*0308***	*0*.*8227*	***0*.*0005***	*0*.*0773*	***0*.*0485***
2019	4.2 ± 1.5 B	13.6 ± 4.1 B	210.1 ± 13.4 A	89 ± 8 A	22, 2 ± 2.9 A	0.66 ± 0.01 A	13.1 ± 0.3 A	3.4 ± 0.1 B
2020	5.9 ± 1.5 A	19.5 ± 5.1 A	212.2 ± 18.4 A	86 ± 10 A	21.7 ± 2.7 A	0.64 ± 0.12 A	10.7 ± 0.5 B	3.9 ± 0.2 A
*p-value*	***0*.*0002***	***0*.*0002***	*0*.*6521*	*0*.*3207*	*0*.*6316*	*0*.*4562*	***<0*.*0001***	***<0*.*0001***
Treatment * YEAR	*0*.*5266*	*0*.*5266*	*0*.*5076*	*0*.*9043*	*0*.*9926*	***0*.*0001***	*0*.*1593*	*0*.*9559*
p-value								

**Fig 1:**
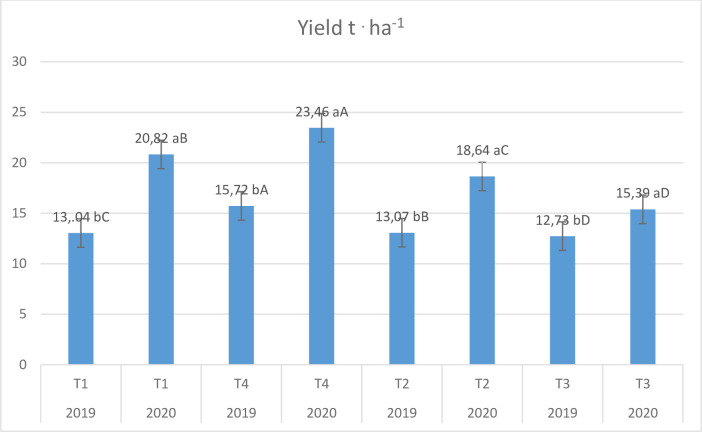
Yield of ´Bluecropˋ highbush blueberry in the subsequent years of research depending on the fertilization technologies used. Error bars indicate standard error.

The yield of highbush blueberries in the subsequent years of research ranged from 12.7 to 23.5 t^.^ ha^-1^. In the first year, the bushes treated with the implementation preparation (T3) yielded the lowest, reaching 12.7 t^.^ ha^-1^, while the best treated with the technology with biostimulation (T4) 15.7 t^.^ ha^-1^, in the case of control (T1) and after the application of the preparation with hormone precursors, the yield was 13.0 t^.^ ha^-1^ (Fig 1). In 2020, the shrubs yielded significantly better than in 2019 (Table 3). In the second year of the study, a similar relation to 2019 was observed in the distribution of the yield of blueberry shrubs (Fig 1). Plants treated with preparations with biostimulation (T4) yielded the best, reaching 23.5 t^.^ ha^-1^, while the least treated with the implementation preparation (T3), reaching the level of 15.4 t^.^ ha^-1^.

The Pearson correlation coefficient of the multivariate analysis was performed for individual treatments, regardless of the year of the study (Table 4). It was shown that in all analyzed treatments, the level of extract in blueberry fruit decreased with the increase in yield, which was confirmed by a significant negative correlation. A substantially positive correlation of firmness with an increase in the yield was demonstrated for the control treatment (T1), with the preparation with hormone precursors (T2) and the implementation preparation (T3). Fruit acidity negatively correlated with the yield, with the increase in the yield, the acidity parameter decreased in the control (T1) and with the use of biostimulation technology (T4). The last crucial correlation in the analyzed parameters is the degree of fruit set. It was found that in the case of the technology with biostimulation (T4) and the implementation preparation (T3), it strikingly negatively correlated with the fruit yield.

**Table 4:** Pearson’s correlation coefficients for multivariate analysis of the impact of fruit yield, t^.^ha^-1^ for blueberry fruit parameters.

Treatments	Mass of 100 berries [g]	Average fruit setting, [%]	Leaf area [cm^2^]	Acidity [% citric acid]	Soluble solids [Brix°]	Firmness [N]
Control–T1	0.18171	-0.23407	-0.29665	**-0.61421**	**-0.79708**	**0.57718**
	0.6154	0.5151	0.4052	**0.0589**	**0.0058**	**0.0806**
Technology with biostimulation–T4	0.26522	**-0.47290**	-0.07315	**-0.41364**	**-0.51991**	0.13284
	0.4589	**0.1675**	0.8408	**0.2347**	**0.1235**	0.7145
Preparation with the precursors of the hormones–T2	-0.30462	0.25847	0.09849	-0.22662	**-0.49771**	**0.64642**
	0.3921	0.4709	0.7866	0.5289	**0.1432**	**0.0434**
Implementation preparation–T3	-0.25648	**-0.49720**	-0.33165	0.30559	**-0.71724**	**0.69862**
	0.4744	**0.1437**	0.3492	0.3905	**0.0195**	**0.0246**

Analyzed irrespective of the treatments used and the year of the study, Pearson correlation coefficient showed a substantial correlation between acidity, extract and firmness in relation to the fruit yield, and showed a strong negative correlation between the extract and firmness (Table 5).

**Table 5:** Pearson’s correlation coefficient for the parameters of yield size and quality.

Fertilizer treatments	Average yield per hectare [t / ha^-1^]	Mass of 100 berries [g]	Average fruit setting, [%]	Leaf area [cm^2]^	Acidity [% citric acid]	Soluble solids [Brix °]	Firmness [N]
Average yield per hectare t / ha	1	0.1787	-0.0672	-0.0954	**-0.4103**	**-0.4530**	**0.3585**
		0.2699	0.6805	0.5580	**0.0085**	**0.0033**	**0.0231**
Mass of 100 berries g	0.1787	1	0.0778	0.0047	-0.2420	-0.0089	0.1272
	0.2699		0.6333	0.9770	0.1325	0.9568	0.4343
Average fruit setting,%	-0.0672	0.0778	1	0.1859	-0.2245	0.2583	-0.0860
	0.6805	0.6333		0.2509	0.1638	0.1076	0.5979
Leaf area cm^2^	-0.0954	0.0047	0.1859	1	0.0517	0.0892	-0.1378
	0.5580	0.9770	0.2509		0.7515	0.5843	0.3965
Acidity% citric acid	**-0.4103**	-0.2420	-0.2245	0.0517	1	- 0.1523	-0.1221
	**0.0085**	0.1325	0.1638	0.7515		0.3482	0.4528
Soluble solids Brix	**-0.4530**	-0.0089	0.2583	0.0892	-0.1523	1	**-0, 7298**
	**0.0033**	0.9568	0.1076	0.5843	0.3482		**<0.0001**
Firmness N	**0.3585**	0.1272	-0.0860	-0.1378	-0.1221	**-0.7298**	1
	**0.0231**	0, 4343	0.5979	0.3965	0.4528	**<0.0001**	

**Fig 2:**
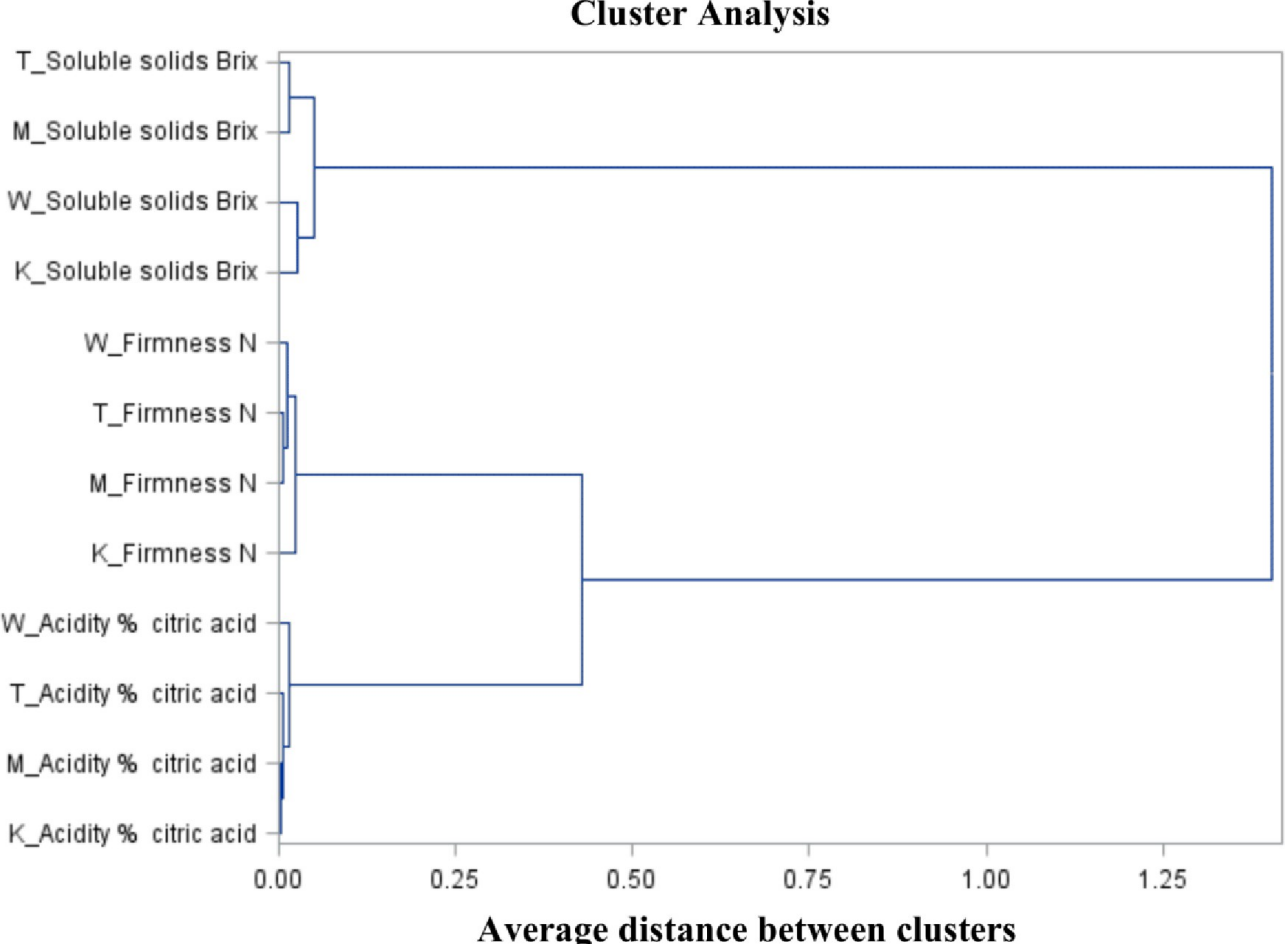
Branching-tree diagram for the extract, firmness and acidity of ´Bluecropˋ highbush blueberry fruit.

Cluster analysis (Fig 2) showed a division into three main clusters, with each cluster relating to individual fruit quality parameters. The first cluster consisting of individual treatmensts of the firmness parameter showed a division into two subsequent clusters, of which the first group is the technology with biostimulation (T4) and the preparation with hormone precursors (T2), whereas the second group is the implementation preparation (T3) and control (T1). In the case of firmness, similarities were demonstrated between the technology with biostimulation (T4), the preparation with hormone precursors (T2) and the implementation preparation (T3), while the deviating object is the control (T1). The acidity parameter showed similarity between the control (T1), the preparation with hormone precursors (T2) and the biostimulating technology (T4), whereas the deviating object was the implementation preparation (T3) (Fig 2).

**Fig 3:**
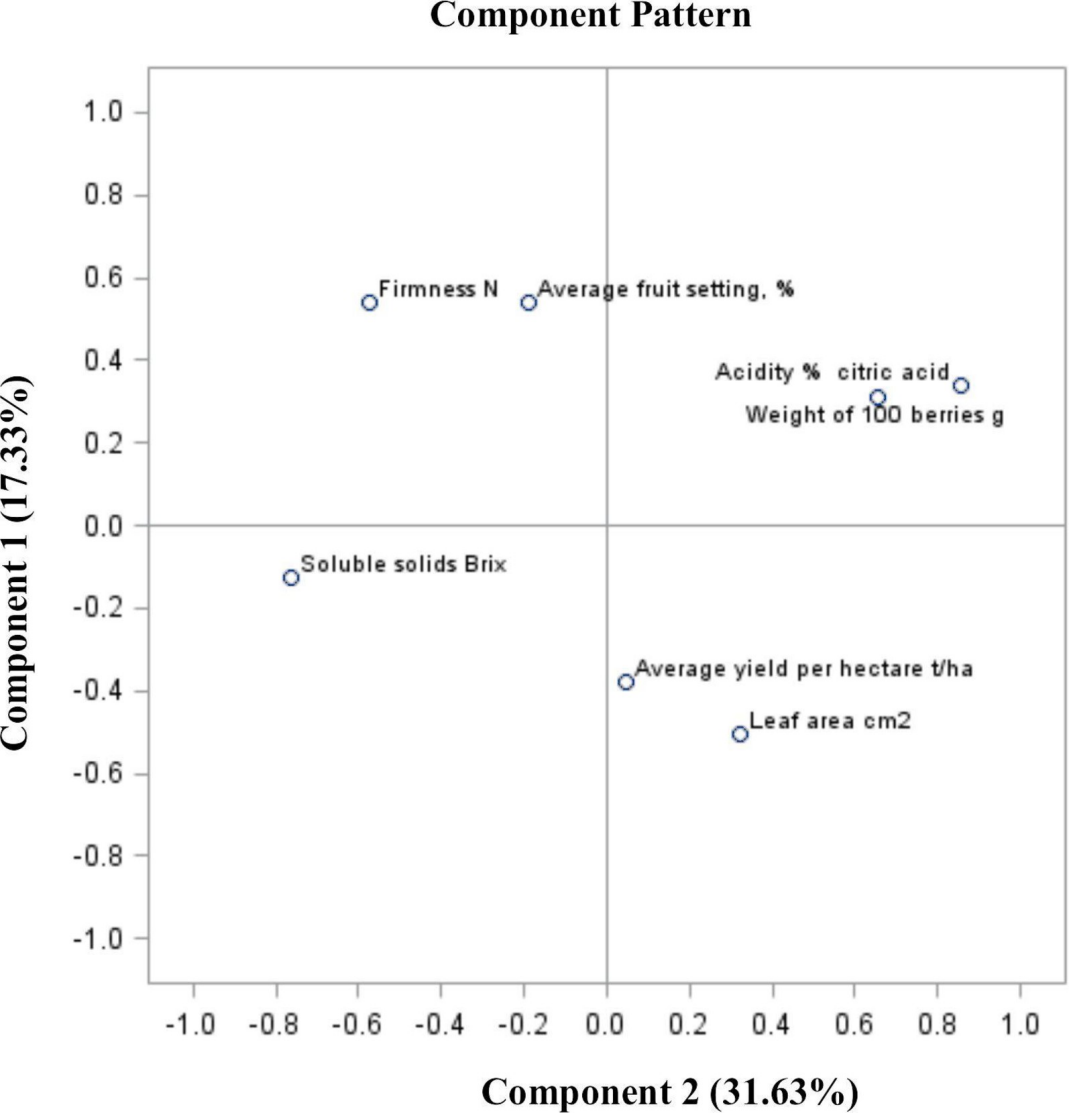
PCA analysis of blueberry fruit in terms of yield size and quality parameters in 2019.

The sum of PC (PC1 and PC2) of the total variable of traits for the fruit of “Bluecrop” highbush blueberry was 48.96% (for PC1 31.63% and for PC2 17.33%, respectively) (Fig 3). When considering the analysis of the parameters of the yield size and quality in 2019, a similarity between specific parameters that formed four groups was observed. The first group is the relationship between the yield and the leaf area, the second is the relationship between the acidity and the mass of 100 berries. The third group is the degree of fruit set and firmness, and the fourth is the extract, which is independent of other parameters (Fig 3).

**Fig 4:**
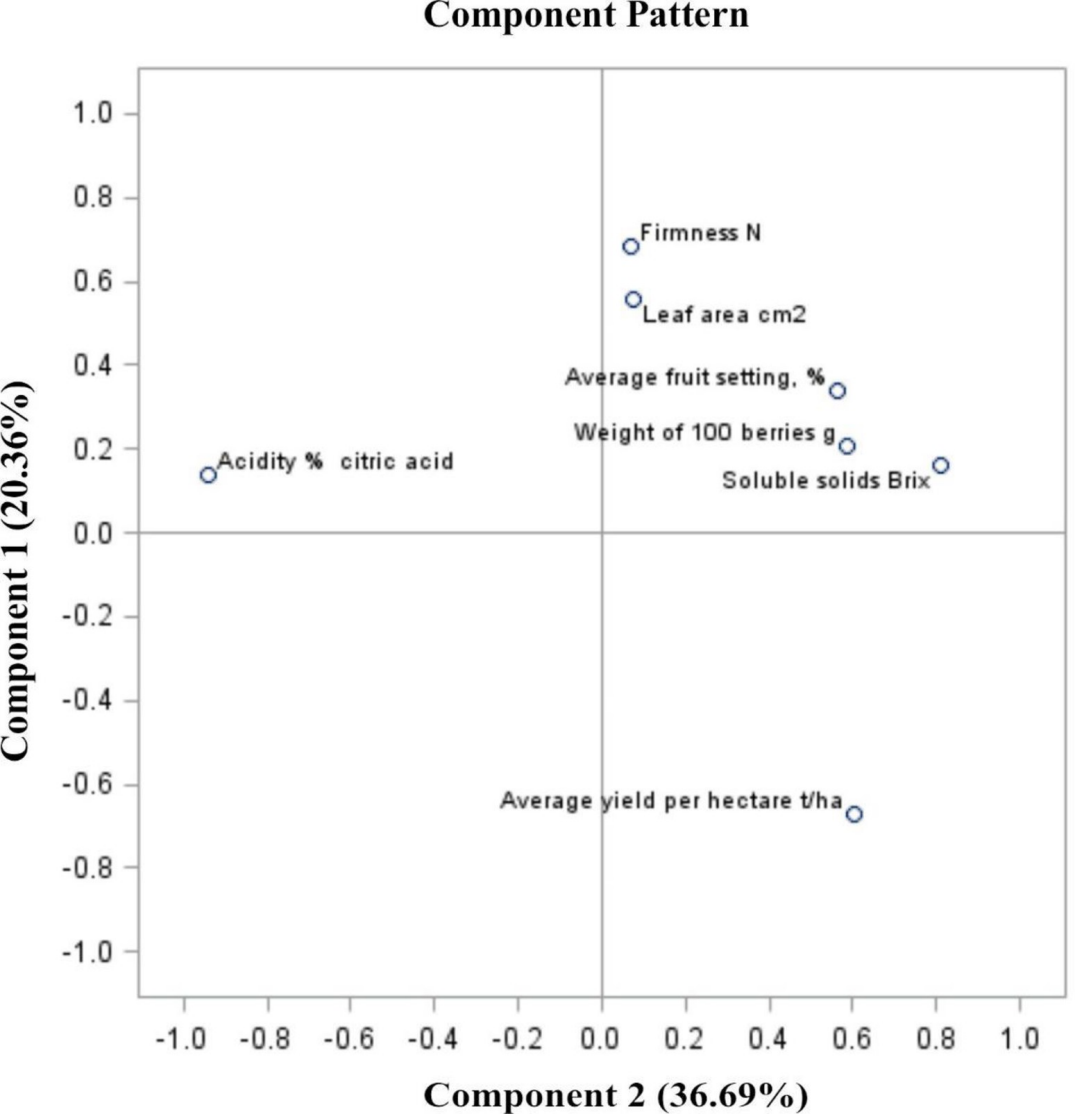
PCA analysis of blueberry fruit in relation to the parameters of the size and quality of the crop in 2020. [component 1, 2].

The sum of the PC of the total variable for the analyzed traits for “Bluecrop” highbush blueberry fruit was 57.05% (36.69% for PC1 and 20.36% for PC2, respectively) (Fig 4). When analyzing the assessed parameters in 2020, three groups of clusters were found. The first and second are independent parameters: acidity and yield. The third and largest group of dependent features are firmness, leaf surface, degree of fruit set, mass of 100 berries and extract (Fig 4).

## Discussion

Bioactive substances influence several physiological processes taking place in the plant at the cellular level. This improves the efficiency and functioning of the entire plant organism [39]. There are many studies in the literature showing the positive effect of biostimulation preparations on the overall biological performance of plants [40, 41]. The above dependence was confirmed in the present study, where the biostimulating program had a positive effect on the yield and the degree of fruit set in comparison to the other treatments, in the case of the implementation preparation (T3) this effect was significant. This dependence may result from the fact that plants treated with sea algae extracts not only take up and assimilate nutrients faster compared to untreated ones [22], they are also characterized by more robust and stronger growth [9, 21] and finally, have a well-developed root system with numerous small side roots [23, 24] capable of wider nutrient uptake. Ohta et al. (2004) [42] showed that phytoregulators used around flowering, improve flowering efficiency, flowers vitality, pollen vitality and quality, and the efficiency of the pollination and fertilization process, which directly translates into improved fruit quality and quantity. Abetz and Young (1983) [43], Featonby-Smith and Van Staden (1987 a, b) [44, 45], Arthur et al. (2003) [46] report that applications of preparations based on sea algae extracts contribute to earlier flowering, better setting and fruit development of many crops. Also, in the research presented in this paper, the positive effect of biostimulating preparations on the setting and mass of the tested fruit was proved. The degree of fruit set in shrubs treated with the biostimulating program (T4) was the highest among all the assessed treatments, compared to the implementation preparation (T3), this effect was significant. A substantially better degree of fruit setting in treatment T4 is associated with the beneficial effect of sea algae extracts on reducing biotic and abiotic stresses—on the effectiveness of plant protection against diseases and pests [28, 29], drought tolerance [30] and high temperature [31–33]. The size of the fruit determined by the mass of 100 berries in the treatment with the biostimulating program was the highest among all the assessed treatments, these differences were significant compared to the control. Crouch and Van Staden (1992) [47] showed that tomato plants treated with seaweed extracts produced on average 30% larger and better-quality fruit than the control. Similar dependencies were proved in the work of Zadope et al. (2011) [48], foliar fertilizer application with sea algae extracts improved the tomato yield by 5% in relation to the control treatment. The beneficial effect of Ascophyllum nodosum (L.) algae on the size and quality of the grapevine yield of ’Thompson Seedless’ was demonstrated by Norrie and Keathley (2006) [49]. In the course of the three-year study, plants sprayed with sea algae extracts yielded an average of 60,4% better, producing larger fruit and with a greater number of clusters on the bush than the control shrubs. A highly favorable effect of algae on the size and quality of the yield was also shown by: Crouch et at. (1992) [50], Aldworth and Van Staden (1987) [51], Abetz and Young (1983) [43], Featonby-Smith and Van Staden (1983) [45] and Arthur et at. (2003) [46]. The above dependencies were confirmed in this study, and during the research, the beneficial effect of fertilization technology based on sea algae extracts on the total yield was shown, compared to the implementation preparation, this effect was statically significant.

The effect of sea algae extracts on the yield and quality of the crop is related to the presence of bioactive substances, including those of a hormonal nature, such as cytokinins, which are responsible for cell division (Featonby-Smith and Van Staden 1983 a, b) [44, 45]. Nooden and Leopold (1978) [52] report that in plants treated with marine algae extracts, a number of substances responsible for growth and development are shifted from the vegetative organs, i.e. roots, shoots and young growths, to the fruit and used for their development. In studies by Featonby-Smith and van Staden (1983) [45] it was proved that the fruits of tomatoes from the combination treated with preparations based on sea algae have a higher content of cytokinins than tomatoes from the control treatment. The positive effect of the technology with biostimulation, based on seaweed extracts, on the percentage of large fruits, i.e. those with a diameter of more than 7,5 cm, and on the commercial yield in individual years of research, was demonstrated by Kapłan et al. (2013) [53]. Apple trees of the ’Szampion’ variety were assessed. A similar effect of biostimulation was demonstrated in the studies conducted on the ’Golden Delicious’ cultivar. Kapłan (2018) [54] showed in the above experiment the highest share of apples above 7,5 cm in a treatment, where the trees were sprayed four times with the preparation with the Maxifruit phytohormone precursors and the gibberellin program. In addition, the preparation with Maxifruit phytohormone precursors in treatment with the ’gibberellin’ program, similarly to the experiments from previous years, had a very positive effect on the quality and shape of fruit, socket cavities, and the number and size of apple seeds of the assessed apple variety.

The study did not show any significant influence of the applied fertilization programs on the leaf surface of the highbush blueberry. In the studies, Kapłan et al. (2013) [53] found a highly beneficial effect of biostimulants on the leaf surface area of apple trees of the ’Szampion’ cultivar in the second and third years of the study. According to Ferrini and Niceise (2002) [55] the use of biostimulants in Quercus robur seedlings had a favorable impact on the surface and dry mass of leaves but did not affect the fresh / dry leaf mass ratio.

The applied fertilization programs did not have a notable effect on the level of blueberry fruit extract of the “Bluecrop” variety. Similarly, Kapłan et al. (2013) [53], after using the technology based on sea algae extracts in the cultivation of apple trees of the ’Szampion’ variety, they did not show a considerable impact of the above-mentioned technology on the content of the extract. A significant influence of the research year on the assessed quality parameter was demonstrated, and in the first year blueberries had a much higher level of extract than in 2020.The analysis of the blueberry fruit firmness level in this study showed that the assessed parameter highly depended on the applied fertilization technology, the bushes treated with the biostimulation technology (T4) had notably firmer berries than the control ones (T1). The study year also showed a remarkable impact on the assessed fruit quality parameter, and in the first year of the study blueberries were characterized by a strikingly higher level of extract than in 2020. An experiment carried out by Yvin and Dufils (2010) [56] showed that the use of Seactiv technology in the form of three Fertileader Elite applications had no beneficial effect on apple fruit firmness during the harvest of the Pink Lady® Cripps Pink Cov. variety compared with three times the use of calcium chloride. It was found that the fruits treated with Fertileader Elite after three months of storage showed less loss of firmness than the controls. In the experiment of Kapłan et al. (2013) [53], it was presented that the use of the technology with biostimulation had a slight effect on the firmness of apple fruit of the ’Szampion’ variety. The fruit treated with the above-mentioned technology were firmer than the control, but these differences were insignificant. On the basis of this work and published reports, the hypothesis that sea algae extracts have a positive effect on vigor, growth and yielding of plants can be confirmed. This is not only due to a better plant nutrition, but also to a positive physiological reaction to the applied bisotimulation. It is worth noticing that the effect obtained in this study indicates the best parameters obtained in the T4 combination, where a full biostimulating program was applied. In combinations where only single biostimulation complexes were used, such spectacular increases were not observed, e.g. for the yield (T2, T3). This allows the conclusion that the various sea algae extracts work synergistically with each other.

## Conclusion

Based on the conducted research, it was proved that the applied fertilization technologies had a significant impact on the size and quality of the yield of “Bluecrop” highbush blueberry. Particularly noteworthy is the fertilization technology based on biostimulating products, which has shown a beneficial, but not always considerable effect on the yield, fruit mass, degree of setting and firmness of the berries. The applied fertilization technologies had no serious effect on the leaf surface and the level of blueberry fruit extract. The performed statistical analysis showed a major influence of the year of the research on the yield, extract level and fruit firmness, in the case of other parameters, the above relationship was not observed.
